# Comparison of the procoagulant activity between extracellular vesicles obtained from cellular monolayers and spheroids

**DOI:** 10.1007/s11239-025-03076-4

**Published:** 2025-03-05

**Authors:** Araci M. R. Rondon, Sophie Featherby, Tainá Gomes, El Houari Laghmani, Camille Ettelaie, Henri H. Versteeg

**Affiliations:** 1 Einthoven Laboratory for Vascular and Regenerative Medicine, Division of Thrombosis and Hemostasis, Department of Internal Medicine, Leiden University Medical Center, Albinusdreef 2, 2333 ZA Leiden, The Netherlands; 2 Biomedical Section, University of Hull, Kingston Upon Hull, UK

**Keywords:** Thrombosis, Neoplasms, Blood Coagulation, Extracellular Vesicles, Microvesicles

## Abstract

Tissue factor (TF) is the main activator of blood coagulation and is associated with thrombosis and tumor progression. It can be released into the blood circulation incorporated within cancer cell-derived extracellular vesicles (EVs). In this study, we investigated the influence of two-dimensional (monolayer) and three-dimensional (spheroid) tumor cell culture methods, and co-culture with cancer-associated fibroblasts (CAF), on the level of EVs release and the associated TF release and activity. The density of EVs and TF released from spheroids and monolayers of Hs578t human breast cancer and CAF were measured by the concentration of the phosphatidylserine and TF-ELISA. For some experiments, cells were activated using a protease-activated receptor (PAR)-2-activating peptide (PAR2-AP). The concentration and EV’s size were accessed by nanoparticle tracking analysis, and a clotting assay was used to evaluate TF pro-coagulant activity. Hs578t monolayers released sevenfold more EVs, and it was associated with an 11-fold higher TF antigen release than the spheroids cultures. Activation of the cells with a PAR2-AP resulted in a significant increase in the release of EVs and TF from the Hs578t monolayers, but no significant increase was observed in the spheroids, only from half Hs578t, half CAF spheroids. Taken together, our results demonstrate that monolayer cell cultures are capable of releasing more significant amounts of EVs and associated TF than spheroid cultures. Monolayers and spheroids have different behavior when we compare the release of EVs and TF. It is essential to consider it when choosing a cell model to study cancer-associated thrombosis.

**Graphical Abstract:**

Generation of extracellular vesicles (EVs) and release of tissue factor (TF), the initiator of coagulation. This was studied after growing cells in monolayers or more physiological spheroids. Monolayer culture cells were shown to release more EVs, and more TF, suggesting that EV and TF shedding using monolayer-based research is not representative of human pathologies such as cancer.

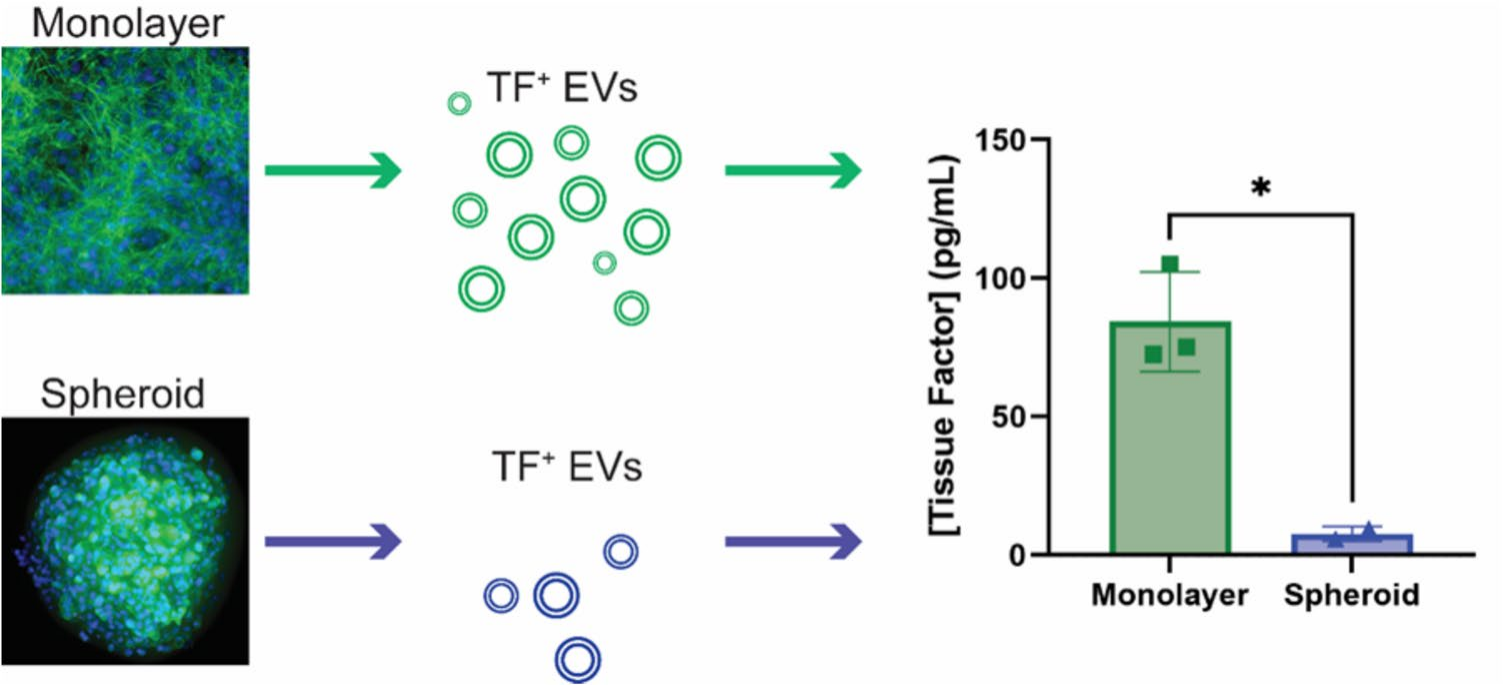

## Highlights

- 2D cultures release more extracellular vesicles with tissue factor than spheroids
- PAR2 activation promotes extracellular vesicles release mainly in 2D cultures
- Co-culture spheroids (of tumor cells and fibroblasts) released more extracellular vesicles
- It is essential to consider the type of cell culture models used in cancer-associated thrombosis research and how accurately they represent human physiology

## 1. Introduction

Cancer-associated thrombosis (CAT) is one of the leading causes of death in cancer patients [1]. CAT has a significant effect on reducing the quality of life and increasing mortality of these patients when compared with patients with only cancer. This research field’s main challenge is finding a better model to investigate the mechanisms involved with this pathology. Several groups have been working with mice models in the last decades, although these are not ideal. Mice cannot develop thrombosis without human manipulation, as they are not affected by the same risk factors that humans are exposed to, such as aging, cancer, anticonception and chemotherapy [2–4].

Extracellular vesicles (EVs) are secreted from the endosomal compartment or the plasma membrane of cells in the form of exosomes and microvesicles, respectively. EVs can promote communication between cells by transporting proteins, lipids, and nucleic acids (e. g. mRNA, RNA, and DNA) [5]. One of the first proteins described to be incorporated in EVs was tissue factor (TF), the main activator of blood coagulation. TF is associated with thrombosis, tumor progression and it may be released into the blood circulation incorporated within cancer cell-derived EVs. Indeed, several in vitro studies have shown that cancer cells shed EVs containing procoagulant TF [6, 7] and clinical studies have suggested that TF-containing EVs are associated with and may cause thrombosis in cancer patients [8, 9], but this view is disputed by others [7, 10]. It is well possible that the inconsistencies between in vitro studies on the one hand and clinical studies on the other stem from the cellular models used to study TF-EV shedding.

Two-dimensional (2D) cell culture models have been used for the last 100 years. Although most in vitro research is performed using cell monolayers, it has been recognized that 2D models do not truly represent the three-dimensional (3D) environment in organisms. 3D culture spheroids consist of a more complex structure as there is cell–cell interaction in multiple dimensions and direct communication with the extracellular matrix. Recently, some research groups have been focusing their attention on 3D models that could better reproduce the secretion of EVs. For instance, EVs containing small RNA derived from 3D cell culture more closely resembled EVs derived from cervical cancer patients plasma [11].

To explain the discrepancies between current in vitro models to generate procoagulant EV and outcomes of clinical studies, we have here compared TF-EV shedding in 2D models with shedding in a 3D model that more closely resembles tissue architecture in vivo.

## 2. Materials and methods

### 2.1. Cells culture

The breast cancer human cell line Hs578t (American Type Culture Collection, ATCC, Virginia, United States) and the breast cancer-associated fibroblast (CAF) were cultivated in Dulbecco’s modified Eagle’s medium (DMEM, 41,966,029, Gibco, Thermo Fisher, Massachusetts, United States) containing 10% fetal bovine serum (FBS) and 1% penicillin (100 U/mL) and streptomycin (100 µg/mL) at 37 °C in 5% CO_2_ atmosphere. CAF cells were kindly donated by Prof. Charlotte Kuperwasser (Tufts University School of Medicine, Boston, US). Cell lines were tested routinely for mycoplasma contamination using MycoAlertTM (LT07-118, Lonza).

### 2.2. 2D and 3D cell culture models

Spheroids of Hs578t and CAF were generated using 10,000 cells/well, which were seeded out in ultra-low attachment round bottom 96-wells plates (Corning, Merck, Germany) and cultured for 3 to 4 days. Parallel sets of cell monolayers were prepared alongside using an equal number of cells seeded in adherent flat bottom 96-wells plates.

### 2.3. Immunofluorescence

Spheroids or monolayers were washed with phosphate-buffered saline (PBS), fixed with 4% paraformaldehyde for 15 min, permeabilized with 0.3% Triton X-100 (Art11869, Merck, Germany) for 15 min. Afterward, cultures were stained with 50 µg/mL Phalloidin fluorescein isothiocyanate labeled (P5282, Merck) for 45 min and Hoechst 33,258 diluted 1:1000. All solutions were diluted in PBS. Imaging was done using an ImageXpress Micro confocal microscope (Molecular Devices, USA) and processing by FIJI [12] or Imaris for 3D reconstruction (Oxford Instruments, UK).

### 2.4. qPCR

RNA samples from monolayers and spheroids were made using TRIsure (BIO-38033, GC Biotech, UK) following the manufacturer’s specifications. cDNA was synthesized using 1 µg RNA and Invitrogen SuperScript II Reverse Transcriptase (Thermo Fisher Scientific, USA). And qPCR was performed using primers (Table 1) and SYBR Select Master Mix (Thermo Fisher Scientific, USA) on a CFX384 Touch Real-Time PCR detection system (Bio-Rad, USA).

**Table 1:** Primer sequences used for qPCR

Primers	Forward sequences	Reverse sequences
*TF*	5’-TACAGACAGCCCGGTAGAGT-3’	5’-AGCTCCAACAGTGCTTCCTT-3’
*PAR2*	5’-TGCTTGTGGTGCATTATTTTCTG- 3’	5’-GGGCTACAATGTACAGGGCATAG-3’
*GAPDH*	5’- TTCCAGGAGCGAGATCCCT-3’	5’- CACCCATGACGAACATGGG-3’

### 2.5. EVs quantification

Cells were washed two times with PBS and pre-adapted to serum-free DMEM for 1 or 2 h. The density of EVs released into the media was measured by evaluating the phosphatidylserine (PS) concentration using the Zymuphen MP-activity kit (#521,096, BioMed, Neuville-sur-Oise, France) according to the manufacturer’s instructions.

### 2.6. TF-ELISA (enzyme-linked immunosorbent assay)

Cells were washed with PBS and serum-free DMEM was added. After 2 h of incubation, cell culture supernatants were collected and centrifugated to remove cell debris at 1,200 g for 5 min. The concentration of the released TF antigen was analyzed using the Quantikine human TF-ELISA (DCF300, R&D systems, Minneapolis, United States) kit according to the manufacturer’s instructions.

### 2.7. Western blot

Cells were seeded in monolayers or spheroids and after 4 days in culture, cells were washed with PBS and 100 µL of 2X Sample Buffer Tris–Glycine SDS (Novex, Thermo Fisher) was added to the wells. Samples were sonicated 10 s at 10 A, heated at 95 °C for 5 min and quantified using nanodrop (Protein A280). 20 µg of samples were loaded in Bolt 4–12% Bis–Tris Plus Gels (Invitrogen) and SeeBlue Plus2 Pre-stained Protein Standard (Thermo Fisher) was used for size evaluation. The proteins were transferred to a PVDF membrane and blocked for one hour at room temperature with 5% milk in tris-buffered saline tween (TBS-T). The membranes were probed with primary antibody: tissue factor (RabMab95, kindly donated by Dr. Vladimir Y. Bogdanov, University of Cincinnati), PAR2 (D61D5, Cell Signaling Technology) and anti-GAPDH rabbit polyclonal (D16H11, Cell Signaling Technology), and incubated overnight at 4 °C. After four washes with TBS-T, HRP-conjugated secondary antibodies (Abcam) were incubated for 1 h at room temperature. The membranes were developed using Western Lightning Plus-ECL (PerkinElmer) and visualized with the ChemiDoc Touch Imaging System (Bio-Rad).

### 2.8. EVs purification and nanoparticle tracking analysis

Cells were washed two times with PBS and serum-free DMEM was added for 2 h. Cell culture medium was collected and centrifuged at 1,000 g for 10 min to remove cell debris. Then, the supernatant was centrifuged 2 times at 20,000 g for 1 h each using 26.3 mL polycarbonate bottles (Beckman Coulter, Woerden, The Netherlands) on a 50.2 Ti rotor using an Optima XE-90 ultracentrifuge (Beckman Coulter). For the second centrifugation, PBS was added to the pellet. The EVs samples were diluted in filtered PBS and measured using a Nanosight NS300 microscope (Malvern, UK). The concentration and size of particles were determined by the analysis of three movies per sample. The temperature was between 19 and 22 °C; the camera was sCMOS. Viscosity was between 0.988 and 0.990 cP, and camera levels were between 13 and 15. The syringe pump speed was 50 AU. EVs that were identified in the PBS were found from the other EVs samples.

### 2.9. Procoagulant activity using clotting assay

A clotting assay was performed to assess the procoagulant activity of EVs using platelet-poor plasma, as previously described [13, 14]. 50 µL of plasma was incubated for 1 min at 37 °C in a coagulometer equipment (STart, Stago, FR). Then, 0.1 ug of EVs from monolayers and spheroids were added and the assay was initiated by adding 100 µL of 6.25 mM CaCl_2_.

### 2.10. Statistical analysis

All experiments were repeated at least three separate experiments with three replicates per group. Results were analyzed by ANOVA and unpaired Student’s t-test using GraphPad Prism 10.2.3 (GraphPad Software, CA, USA). Asterisks indicate a significant difference compared to the control: p < 0.05 (*), p < 0.01 (**), p < 0.001 (***) and p < 0.0001 (****).

## Results

### Comparison of TF and PAR2 expression in 2D and 3D breast cancer cell cultures

**Fig. 1:**
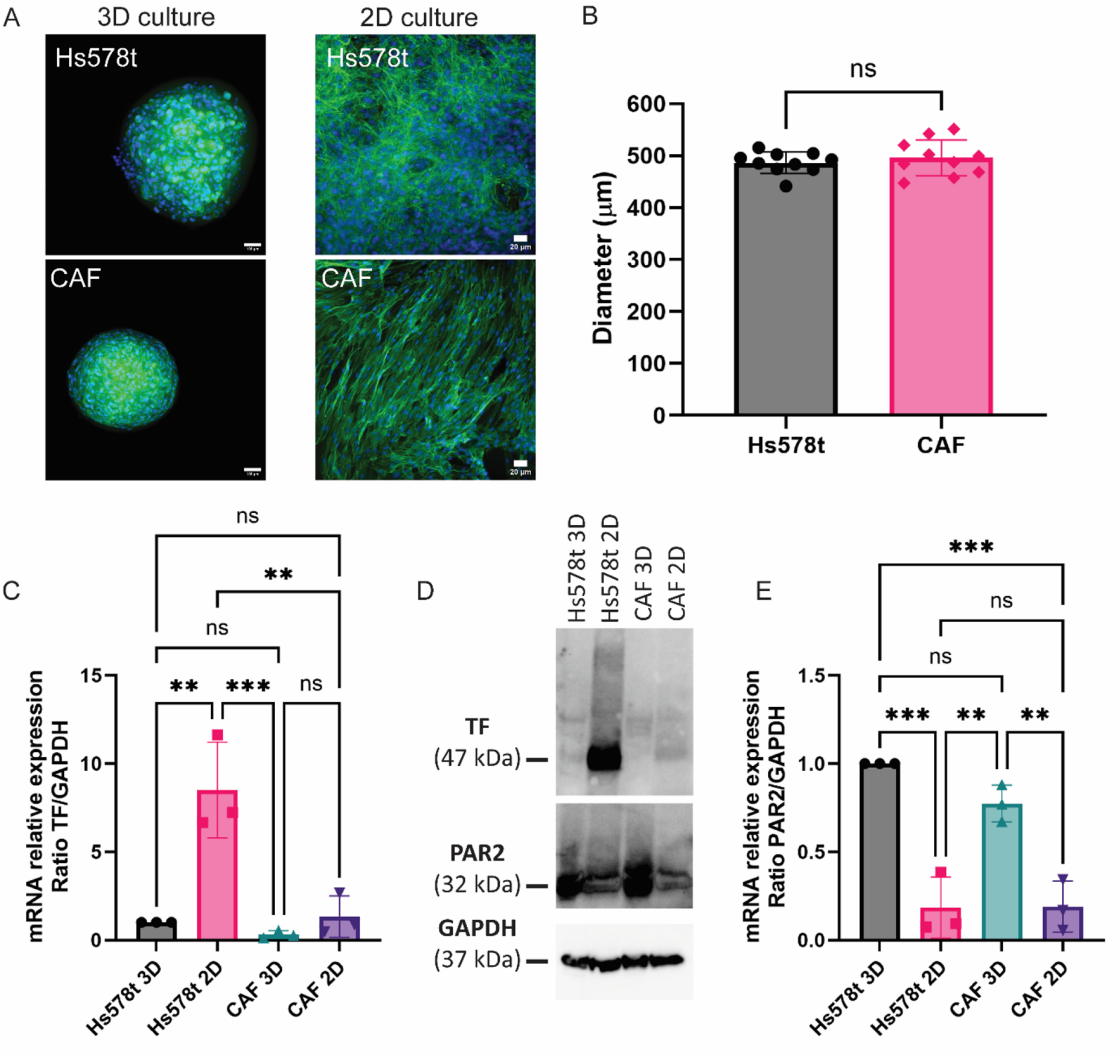
Analysis of cell morphology, TF and PAR2 expression between 2 and 3D cultures. Hs578t and CAF were cultivated as spheroids or monolayers (**A**). Ten thousand cells were seeded in ultra-low attachment round bottom 96-well plates to form the spheroids or in adherent flat bottom 96-wells plates to form monolayer cultures. Bars correspond to 100 µm (3D) and 20 µm (2D). After 3–4 days of culture, spheroids and monolayers were imaged, and the diameter of spheroids was measured (**B**) using FIJI. TF and PAR2 mRNA expression was accessed by qPCR after 3–4 days of culture (**C**, **E**), TF and PAR2 expression was accessed by western blot (**D**) and GAPDH was used as a loading control (n = 3)

To compare shedding of TF-EV in 2D models with that in 3D models, we made use of breast cancer cells, as well as cancer-associated fibroblasts (CAFs) that constitute a key stromal component of the tumor microenvironment. Hs578t is a cell line that represents triple-negative breast cancer, as it lacks estrogen receptor, progesterone receptor, and human epidermal growth factor receptor 2 (HER2). Hs578t or CAF 3D cell cultures were generated by placing them in ultra-low attachment round bottom 96-wells plates (10,000 cells per well). After 3–4 days, the cells formed spheroids with round morphology and diameters of 486,4 µm ± 20,8 and 495,7 μm ± 37,5 (Fig. 1A and B), respectively. 2D cell cultures were prepared alongside by seeding 10,000 cells per well in adherent flat bottom 96-wells plates (Fig. 1A).

TF mRNA expression was higher in 2D cultures of Hs578t when compared to CAF 2D cultures (Fig. 1C). Hs578t cultured 2D showed 8,5 ± 2,7 times more TF mRNA expression when compared with Hs578t 3D cultures; 2D cultures of CAF showed 5,1 ± 4,4 times more TF mRNA expression when compared with CAF 3D culture. Between 2D cultures, Hs578t had 9,0 ± 5 times more TF protein than CAF cultures, as determined by western blot (Fig. 1D). In contrast, mRNA expression of PAR2, the canonical receptor to TF in complex with its binding partner coagulation factor VIIa [15], was higher in 3D cultures when compared with 2D cultures (Fig. 1E). Hs578t cultured in 3D showed 8,9 ± 5,7 times more PAR2 protein than in 2D cultures (Fig. 1D). In addition, 3D cultures of CAF showed 6,8 ± 6,1 times more PAR2 protein expression when compared with CAF 2D culture. In conclusion, 3D culture of cancer cells and CAFs leads to lower TF expression, but higher PAR2 expression.

### Release of EVs and TF from Hs578t 2D and 3D cultures

**Fig. 2:**
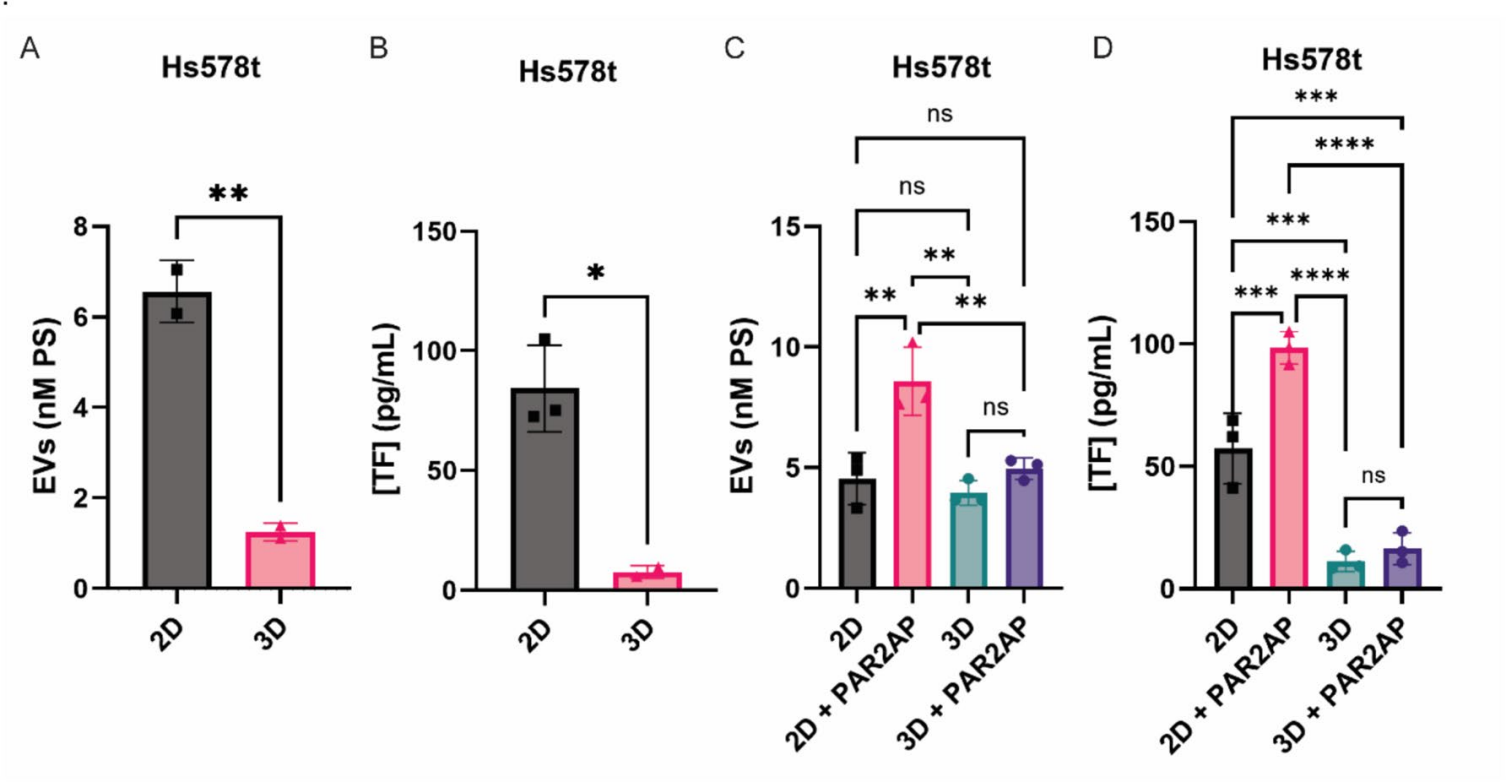
Differences in EV and TF release from Hs578t cultivated in 2D and 3D with and without activation with PAR2-AP. Ten thousand cells were seeded in ultra-low attachment round bottom 96-well plates to form spheroids or in adherent flat bottom 96-wells plates for the monolayer culture. After 72 h, the culture supernatant was removed and centrifuged to remove cell debris. The release of EVs (**A**, **C**) and TF (**B**, **D**) from 2 and 3D Hs578t cultures were evaluated in the culture supernatant by Zymuphen MP-activity kit and human TF-ELISA, respectively. One hour of starving was followed by 30 min of activation with 20 µM of PAR2-activation peptide (PAR2-AP, SLIGKV, Fig. 3C, D)

We next studied the release of EVs under 2D and 3D conditions. The release of EVs from Hs578t was sevenfold higher in monolayers compared to the spheroid cultures (Fig. 2A). It was also associated with an 11-fold higher TF antigen release than in the spheroids (Fig. 2B). Three spheroids were pooled for the following experiments to achieve a more comparable parameter for EV release between monolayers and spheroids. It was previously demonstrated that PAR2 activating peptide (PAR2-AP, SLIGKV) promotes EV release and higher TF content on EVs prepared from different cell lines [16]. As shown before, stimulation of Hs578t cultures with PAR2-AP for 30 min in 2D cultures led to increased amounts of shed EVs (Fig. 2C) and TF (Fig. 2D). In contrast, stimulation with PAR2-AP for 30 min in 3D cultures did not result in significant increases in shed EVs and TF (Fig. 2C, D).

### 3D co-cultures of cancer cells and cancer-associated fibroblasts promote release of EVs and TF

**Fig. 3:**
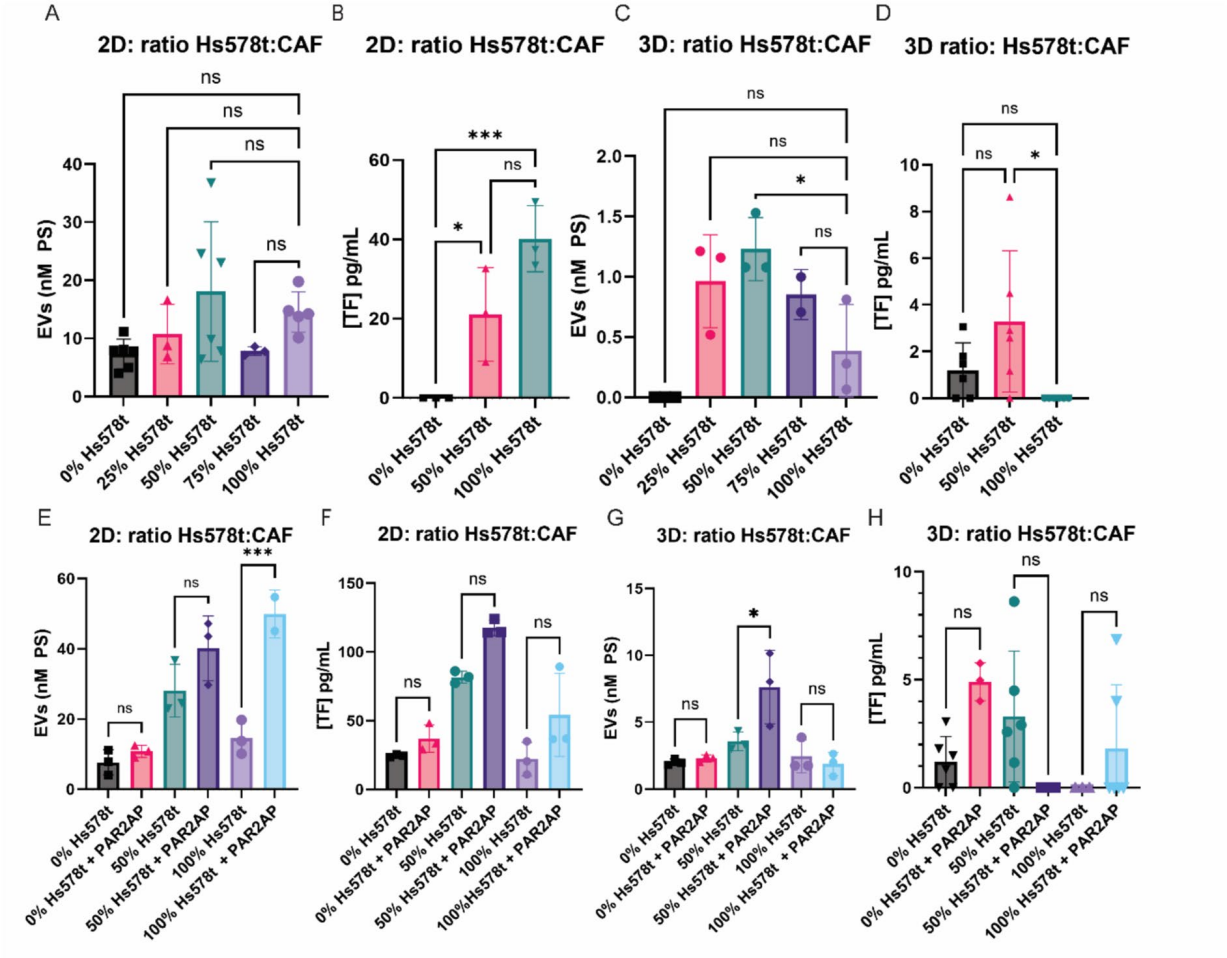
Differences in EVs and TF release from co-culture of Hs578t and CAF. Different proportions of Hs578t and CAF were seeded from 0% Hs578t (100% CAF) to 100% Hs578t (0% CAF) spheroids or monolayer culture. The release of EVs and TF from 2D (**A**, **B**) and 3D (**C**, **D**) co-culture of Hs578t and CAF were evaluated after 72 h in culture followed by serum starvation. The effects of PAR2-AP were also observed in the co-culture from 2D (**E**, **F**) and 3D (**G**, **H**) on the release of EVs or TF

Next, we studied the effects of a pathologically relevant condition, i.e. the presence CAFs, on the shedding of TF-EVs. Hs578t were mixed with CAFs in different proportions to establish 2D and 3D co-cultures of tumor and stroma. CAFs are a critical component of the tumor microenvironment, as this population impacts cancer invasion, tumor growth and drug resistance [17, 18]. As expected, a higher release of EVs from co-cultures composed of 50% Hs578t:50% CAFs was observed under both 2D and 3D culturing conditions compared to 0% Hs578t (Fig. 3A, C). Interestingly, there was also a higher release of EVs from 3D cultures of 50% Hs578t:50% CAFs compared to 100% Hs578t, but it was not significantly higher when compared 2D 50% Hs578t:50% CAFs to 2D 100% Hs578t. Release of TF under 2D conditions was maximal in the Hs578t monoculture, which is expected as Hs578t cells express ninefold more TF than CAFs under 2D culture conditions (Fig. 3B, 1C, D). Unexpectedly, TF release under 3D conditions was maximal when the coculture consisted of 50% Hs578t and 50% CAF (Fig. 3D). These data show that the incorporation of stromal CAFs in cancer cell cultures leads to enhanced TF shedding on EVs, but only in 3D spheroids.

The co-cultures were starved for one hour and stimulated with 20 µM of PAR2-AP for 30 min. The release of EV was increased in 3D 50% Hs578t:50% CAF co-culture (Fig. 3G) compared to the same sample without PAR2-AP stimulation, but not in 2D culture (Fig. 3E). There was a tendency of increasing TF secretion in 2D 50% Hs578t: 50% CAF after PAR2-AP, but not in 3D cultures (Fig. 3F, H).

**Fig. 4:**
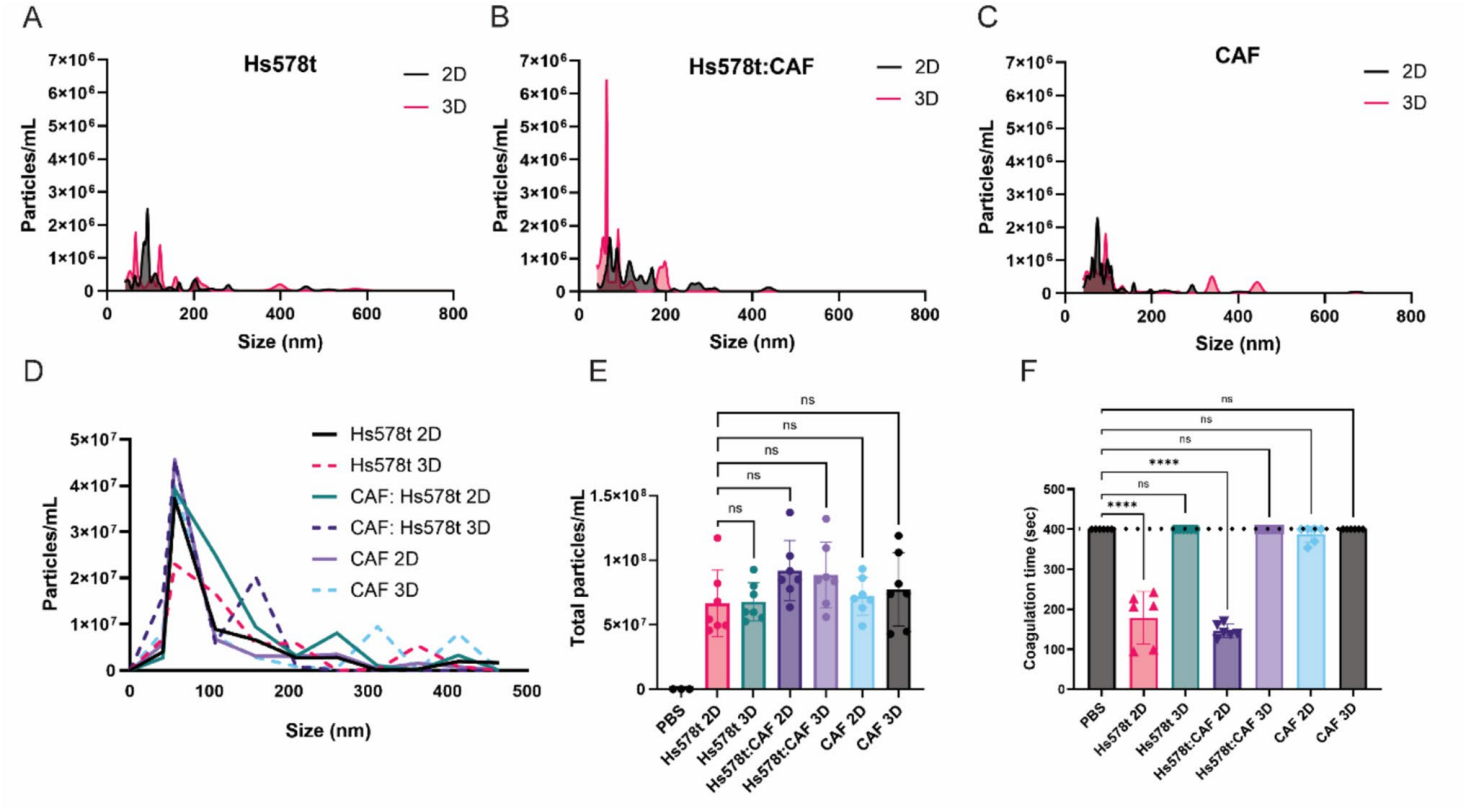
EVs purification and TF activity. After culture for 4 days, cells were washed two times with PBS and DMEM without FCS was added. After 2 h, the medium supernatant was collected and centrifugated at 20,000 g to purify EVs released from cells. The diameter (**A**–**D**) and total particle concentration (**E**) of the vesicles were evaluated by Nanosight. For the clotting assay, 50 µL of plasma was added to a coagulometer equipment (STart, Stago, FR), after 60 s at 37 °C, 0.1 µg of EVs was added to the platelet-poor plasma (**F**). The assay was started with 100 µL of 6.25 mM CaCl_2_(C)

EVs were purified from 96-wells plates and the concentration was evaluated by nanoparticle tracking analysis (Figs. 4A–3E). There was no significant difference between EVs released by 2D or 3D cultures from all cell origins or culture type (Fig. 4E). There was a difference in the size distribution, but most EVs were between 40 and 100 nm. EVs purified from 2D Hs578t or 2D 50% Hs578t: 50% CAF cultures were able to promote an acceleration in coagulation time (Fig. 4F). This was an interesting fact, considering that CAF 2D cultures could not accelerate the coagulation time significantly, but when combined with Hs578t, it caused a reduction in the coagulation time.

## Discussion

Currently, the role of TF-EV in cancer-associated thrombosis is debated. Analysis of the coagulant activity of such EV under in vitro or in vivo conditions shows discrepancies; while TF-EVs show coagulant activity in cell culture setups, such coagulant activity appears limited in the plasma of cancer patients with VTE [6, 7, 10]. We argued that current methods to generate TF-EVs in vitro do not mimic those observed in vivo. To this end, we have employed 2D culture and 3D spheroids to analyze shedding of EVs and EVs-exposed TF under conditions that are relevant to tumor biology. First of all, we observed that monolayer cell cultures are capable of releasing greater amounts of EVs and associated TF than spheroid cultures. This may be explained by the larger surface area that is in contact with medium in the case of 2D cultures compared to 3D culture. This may also explain why the role of TF-EVs in vivo is limited. It may be that TF-EVs only promote VTE in patients with tumors that dramatically overexpress TF, such as pancreatic cancer, but to our knowledge such inter-cancer type analysis on associations between TF expression and risk of VTE has never been performed.

A second unexpected observation pertains to the fact that the presence of CAFs promoted the release of TF in EVs in spheroids, but not in 2D cultures. CAFs are known to influence tumor cell behavior through pathways such as those elicited by e.g. IL-6 [19], HGF/MET [20], IFNβ1 [21] and certain microRNAs [22]. It is currently unknown why CAFs would promote TF release on EVs in a 3D but not 2D setting, but it is tempting to speculate that such pathways are overactivated under 3D conditions where the interaction between tumor cells and CAFs is more intimate.

The final observation we made was that the levels of released EVs and associated TF are more strongly amplified following PAR2 stimulation of monolayer cultures than spheroids. This is surprising as 3D cultures were shown to express more PAR2 than monolayer cultures. We currenty have no explanation for this, but it may be that cells at the periphery of the sphere contain more tight and adherens junctions, preventing PAR2 activation in the center of the spheroid. More experiments on this are warranted.

One open question we have not yet resolved is how many of the EVs shed by a spheroid are derived from the cancer cells and how many are derived from CAFs. Although in theory this could be established by separating the EVs based on cancer cell-specific and CAF specific cell surface marker, in practice this is difficult to establish. In a spheroid, cancer cells and CAFs are in close contact and transfer of proteins via cell–cell contact and EVs does occur as recently shown by Schwager and colleagues [23].

## Conclusions

In conclusion we have shown that spheroids that more closely resemble natural tissue than 2D cell cultures behave differently with regard to EV shedding and procoagulant processes.

## Data Availability

Non-published data are available from the corresponding author upon reasonable request.
